# Effectiveness of Near-Peer Versus Faculty Point-of-Care Ultrasound Instruction to Third-Year Medical Students

**DOI:** 10.24908/pocus.v7i2.15746

**Published:** 2022-11-21

**Authors:** Katie Rong, Grace Lee, Meghan Kelly Herbst

**Affiliations:** 1 Department of Emergency Medicine, Beth Israel Deaconess Medical Center Boston, MA United States; 2 University of Connecticut School of Medicine Farmington, CT United States; 3 Department of Emergency Medicine, University of Connecticut School of Medicine Farmington, CT United States

**Keywords:** Point of Care Ultrasound, medical student, Medical Education, near peer education, ultrasound curriculum

## Abstract

**Background: **Incorporation of point-of-care ultrasound (POCUS) in undergraduate medical education (UME) is expanding; however, its effective implementation is impeded by a lack of trained faculty. Recruitment of near-peer (NP) instructors is a potential solution, but there are concerns surrounding NP teaching effectiveness compared to faculty instruction. While some institutions have assessed supplemental NP instruction, or NP-taught sessions with strict faculty supervision, few if any have compared effectiveness of NP POCUS instruction alone to faculty instruction through a multi-dimensional assessment. The aim of this study was to compare the effectiveness of near-peer (NP) instruction to faculty instruction at an undergraduate medical education clinical POCUS session for third-year medical students. ** Methods:** This was a randomized controlled trial where third-year medical students were assigned to one of two groups for a 90-minute POCUS session: NP instruction or faculty instruction. A pre- and post-session multiple-choice test and a post-session objective structured clinical examination (OSCE) were administered to assess conceptual and hands-on clinical POCUS knowledge gained. Students’ perceptions of the instructors and session were evaluated using a Likert scale. **Results**: Seventy-three students (66% of the class) participated; 36 taught by faculty and 37 by NP instructors. Both groups showed a significant score increase from pre-test to post-test (p =0.002); however, there was no significant difference between groups in post-test (p=0.27) nor OSCE scores (p=0.20). Student perceptions of instructor competency were not statistically significant. **Conclusions**: NP instructors were as effective at teaching clinical POCUS to third-year medical students as faculty instructors at our institution.

## Background

Implementation of point-of-care ultrasound (POCUS) in undergraduate medical education (UME) has increased due to a growing appreciation for its value to students as a tool for learning anatomy, enhancing data acquisition during the physical exam, and formulating a diagnosis 1. Although the American Institute of Ultrasound in Medicine lists over 200 medical schools in the United States as having POCUS education, most curricula are very new, fragmented, or ineffective at meeting the intended objectives 1.The limited number of clinical faculty available to teach is one of the most commonly cited obstacles 1, 2. 

Achieving small student-to-instructor ratios for skill-based learning results in more personalized and effective instruction essential for teaching POCUS 3, 4.However, medical schools generally find this challenging due to financial and resource constraints, difficulty securing enough trained faculty to teach, and unpredictable scheduling and teaching quality inconsistencies when recruiting on a volunteer basis 4, 5, 6. One potential solution is the use of near-peer (NP) teaching. 

NP teaching is defined as students or resident physicians teaching students at an earlier phase in their medical school education or training continuum 7. In our study, we defined NPs as fourth-year medical students or medical residents. Skepticism around NP teaching revolves around fear that students given NP instruction will receive inferior education due to their inexperience in teaching 8.Studies have attempted to counter this skepticism by demonstrating the value of NP teaching using simple POCUS applications^9^, ^10^ and non-ultrasound skills^4^, ^11^, ^12^ as educational content. Additionally, studies have demonstrated improved NP instructor confidence and learner comfort in a setting where they share more social and cognitive congruence with their instructors 13, 14, 15. Still, persistent apprehension has limited research efforts to study NP effectiveness, and many studies include safety measures such as NP teaching sessions serving only as supplemental instruction to faculty teaching^13^, ^16^ or NP instructors being actively supervised by a faculty member while they are teaching 13.

Our study aimed to compare effectiveness of NP to faculty POCUS instruction across multiple clinical applications during a required third-year medical student session. Effectiveness was measured by the students’ post-session objective structured clinical examination (OSCE) scores, post-session multiple-choice knowledge assessment scores, and improvement in score from pre-session to post-session multiple-choice knowledge assessment. We also aimed to assess student perceptions of NP and faculty instructors, measured by a validated 5-point Likert scale. 

## Methods

### Study protocol 

This was a randomized prospective study of third-year medical students that took place during a four-day curriculum called “Homeweek” at the end of October 2020. At the time of the study, students had already received approximately 20 hours of POCUS instruction across their first two years of the undergraduate medical education (UME) curriculum, with some students having more experience through electives or shadowing. All students provided written consent for the study. No financial compensation was provided for participation. Students were told explicitly in person and in writing that participation in the study will have no effect on their course grade. The study was approved by the site Institutional Review Board. 

### Recruitment

Students were randomly assigned to one of two groups, faculty group or NP group, based on the alphabetical order of the students’ last names. Students were blinded to their group assignment. During the teaching sessions, all instructors introduced themselves by their first name only in order to blind their training level to the students. Faculty instructors were defined as attending physicians with ultrasound fellowship training or more than ten years of ultrasound experience and consistent utilization. Faculty instructors were recruited from multiple hospital emergency medicine and critical care departments through which medical students do not rotate until they are in their fourth year. NP instructors were defined as experienced fourth-year medical students and emergency medicine residents. Experienced fourth-year medical students were defined as those who completed at least 2 weeks of elective formal ultrasound training in addition to the required vertical ultrasound curriculum offered by the medical school. All instructors were recruited on a voluntary basis and given a detailed instructor guide to ensure consistency in the objectives covered (Supplementary Material Appendix A). None of the instructors were given any in-person training on how to teach or cover the objectives. No financial compensation was provided for participation.

Prior to the sessions, third-year emergency medicine residents proficient in POCUS but not participating as NP instructors during Homeweek were asked to pilot either the pre- or the post-test questions to establish face validity (residents invited to provide feedback on clarity of questions and answer choices) and criterion validity (resident scores expected to be greater than 80% for both pre- and post- test). Each pre-test question had a paired post-test question pertaining to basic ultrasound knowledge or clinical ultrasound application (Supplementary Material Appendix B) to ensure that the content of both evaluations covered similar topics in the same proportions. 

### Ultrasound Curriculum 

Homeweek POCUS sessions covered six applications. Two applications were required to solve each of three clinical vignettes in 90 minutes. The three paired applications were: 1) ocular and thoracic, 2) cardiac and shoulder, and 3) biliary and renal. Students spent 25-30 minutes with each instructor for each paired application, interacting with three different instructors of the same status (all faculty or all NP) by the end of the session. No students were exposed to a mix of NP and faculty instructors. Instructors read the clinical vignettes and asked students to assess the etiology of the patient’s complaint using POCUS on the volunteer live model. Instructors provided feedback on students’ technique, reviewed pertinent sonoanatomy, and once adequate views were obtained, instructors demonstrated what sonopathology they would have seen if scanning the patient featured in the vignette, using prepared slides with video clips. 

### Data Collection

Before the ultrasound session, all third-year medical students took the Pre-Session Evaluation (pre-test) via online survey (Supplementary Material Appendix C), which consisted of ten multiple-choice questions. Students additionally were asked if they had any ultrasound experience outside the standard medical school curriculum. Students with no extra ultrasound experience were given an experience score of 0 points while those with any extra experience were given 1 point. The purpose of this score was to confirm randomization of students with prior ultrasound experience across NP- and faculty-taught groups.

All students completed a 15-question Post-Session Evaluation (Supplementary Material Appendix D), and a ten-minute OSCE. The Post-Session evaluation consisted of ten questions covering general ultrasound knowledge (referred to in the results section as the “post-test”), followed by five Likert scale questions asking students to rate their confidence in performing POCUS, each instructor’s competency, whether the instructor was able to create a comfortable learning environment, and the organization of the session. The pre- and post-session multiple-choice questions were written by the ultrasound director and were piloted by third-year emergency medicine residents who did not participate as NP instructors. Questions were tailored from Likert survey questions previously validated (Cronbach alpha) and used in the assessment of NP instructors in other studies 17. The OSCE was modified from others previously published according to structures taught during the Homeweek session 13. During the OSCE, students were given ten minutes to find 15 structures on a human model using ultrasound, and to limit variation, OSCE proctors were given the OSCE Instructor Guide (Supplementary Material Appendix E). 

### Statistical Analysis

For the mean post-test scores between NP- and faculty-taught students, we assumed a 1.5-question difference to be of academic significance with a predicted standard deviation of 2 questions. With this assumption, a minimum of 29 students per group were needed to detect 80% power at the 0.05 significance level for the difference in mean using a two-sided two-sample t-test. Similarly, we assumed a 2-question difference in the mean OSCE score between groups to be of academic significance, with a predicted standard deviation of 2.5 identified structures. With this assumption, a minimum of 26 students per group were needed to detect 80% power at the 0.05 significance level for the mean difference using a two-sided two-sample t-test.

For the ultrasound experience score, pre- and post-test, and OSCE, the mean scores of the students taught by NP instructors were compared with those taught by faculty instructors using a two-sample t-test. To assess for improvement after the sessions, the change in pre-test and post-test scores (ΔPrePost) was calculated and then the two groups were compared using a two-sample t-test. A Wilcoxon Rank Sum test was used to analyze the Likert scale data ranging from strongly disagree (1) to strongly agree (5). Statistical analyses were performed using SPSS statistical software, version 28.0.0.0 (SPSS Inc., Chicago IL) and Microsoft Excel (2016).

## Results

### Evaluation and Randomization Validation 

Fifteen emergency medicine residents participated in piloting the pre- and post-test questions. Seven residents took the pre-test and received a mean score of 8.43 (SD ±0.53) while 8 residents took the post-test for a mean score of 8.13 (SD ±1.25). The difference in scores was not statistically significant (P=0.56) and thus we assumed the two evaluations were of similar difficulty and a valid measurement in assessing for knowledge acquisition from the sessions. 

Seven faculty and 11 NP instructors taught six sessions. Four faculty were ultrasound fellowship-trained emergency physicians; one faculty was an ultrasound fellowship-trained pediatric emergency physician; one faculty was trained in internal medicine and completed a fellowship in critical care; one faculty was an emergency physician with over a decade of significant ultrasound experience. Of the 11 NP instructors one was a third-year resident in emergency medicine, two were second-year residents in emergency medicine, five were first-year residents in emergency medicine, and three were fourth-year medical students who completed an ultrasound elective in medical school and showed interest in POCUS outside of the curriculum. 

Of the entire third-year class (110 medical students) invited, 73 (66%) students consented to participation in the study with 36 randomized to the faculty group and 37 randomized to the NP group. Adequate randomization was demonstrated by a lack of statistical significance (P=0.06) between the faculty and NP group in mean ultrasound experience scores (faculty 0.33 points, NP 0.24 points) and mean pre-test scores (faculty 4.67 questions correct, NP 5.54 questions correct). 

### Knowledge Assessment and OSCE

While students taught by NP-instructors scored higher on the pre-test and post-test, and students taught by faculty instructors scored higher on the OSCE, there was no statistically significant difference in the mean post-test scores (faculty 6.14 questions correct, NP 6.65 questions correct; P=0.27) or in the mean OSCE scores (faculty 6.94 questions correct, NP 6.11 questions correct; P=0.20). Both groups had a statistically significant increase in mean scores from the pre-test to the post-test (P=0.002), but the difference in increased mean score was not statistically significant (P=0.52). See Table 1.

**Table 1:** Comparison of Mean Pre-test, Post-test, OSCE, and ΔPrePost scores for 36 Faculty-taught students vs. 37 NP-taught students

	Faculty Instructors (n=36) Mean (SD)	NP Instructors (n=37) Mean (SD)	Difference 95% CI	P-value
Pre-test ^a^	4.67 (1.97)	5.54 (1.87)	[-1.77, 0.02]	0.06
Post-test ^a^	6.14 (2.10)	6.65 (1.83)	[-1.43, 0.41]	0.27
OSCE ^b^	6.94 (2.98)	6.12 (2.47)	[-0.44, 2.11]	0.20
ΔprePost ^c^	1.47 (2.66)	1.11 (2.07)	[-0.74, 1.47]	0.52
Difference 95% CI	[0.57, 2.37]	[0.42, 1.80]		
P-value	0.002	0.002		
Abbreviations: SD, standard deviation; NP, near-peer; OSCE, objective structured clinical examination; ΔPrePost, change in pre- and post- test scores ^a ^Pre-test and post-test involved a ten-question multiple choice evaluation ^b ^Mean number of correctly identified structures in a 15-question OSCE ^c ^ΔPrePost reflects the increase in mean number of questions correctly answered in the ten-question multiple choice evaluation after the educational session				

### Student Perceptions of Instructors 

The mean Likert scale scores of students’ perceptions of instructor competency and ability to create a comfortable environment were higher in the faculty group compared to the NP group. In contrast, the mean score for the NP group’s improved confidence with POCUS was higher compared to that of the faculty instructor group. However, these differences were not statistically significant. See Table 2.

**Table 2:** Student perceptions 36 Faculty-taught students vs. 37 NP-taught students using a Likert scale ranging from strongly disagree (1) to strongly agree (5)

Question^ a^	Faculty Instructors Mean (SD)	NP Instructors Mean (SD)	P- value
Instructor for the first session was competent.	4.69 (0.47)	4.57 (0.5)	0.39
Instructor for the first session created a comfortable environment.	4.71 (0.46)	4.49 (0.56)	0.13
Instructor for the second session was competent.	4.60 (0.50)	4.51 (0.51)	0.53
Instructor for the second session created a comfortable environment.	4.60 (0.50)	4.51 (4.51)	0.93
Instructor for the third session was competent.	4.66 (0.48)	4.43 (0.65)	0.21
Instructor for the third session created a comfortable environment.	4.63 (0.48)	4.46 (0.73)	0.51
My confidence in performing point of care ultrasound has improved through participation in the ultrasound session.	4.14 (0.81)	4.27 (0.61)	0.70
Abbreviations: NP, near-peer ^a ^Students within the faculty and NP groups each had three separate instructors for the three paired applications covered in the educational session			

## Discussion

This is the largest randomized study to our knowledge that compares effectiveness of NP and faculty POCUS instruction across multiple clinical applications through multiple objective and subjective assessments 9.In a recent study assessing the state of UME POCUS training in America, only a small number of medical schools had a longitudinal curriculum; citing the most common barrier being lack of trained faculty. Robust data is necessary as POCUS education becomes more widely taught 1, 2, and our study supports the use of NP instructors as an effective tactic to maintain the high quality of education while working within resource constraints. 

Medical students in their clinical years (typically years 3 and 4) are trained to collect and interpret data according to clinical context. POCUS instruction at this level of training needs to match these objectives. This is the first study comparing NP and faculty POCUS instruction that is case-based, patient-oriented, and goes beyond a single application to address a patient complaint. Despite the complexity of these sessions, our data mirror conclusions of prior single-application studies, supporting the effectiveness of NP instruction.

The comprehensive study design, similar to Knobe et al. but with the addition of a pre-test to control for prior ultrasound experience, strengthen our results and conclusion 9. We attempted to limit confounding variables to accurately and comprehensively evaluate the effectiveness of instruction. Another strength of the study is the small student-to-instructor ratio, capped at 3:1 given skill-based learning is most effective near this ratio  3, 4. Previous studies that demonstrate NP instruction effectiveness have a student-to-instructor ratio greater than 5:1, which may have had a negative impact on instructor effectiveness confounding results 9, 10, 12. It is assumed that all participants were given adequate time to perform a full ultrasound examination during each hands-on session. However, it must be stated that we did not confirm that this was the case. While we estimated that timing should not be an issue when planning the sessions, the efficiency of an instructor is a factor in their effective teaching skills and presumably would be reflected in the post-session evaluations.

While well-powered, a major limitation of this study was incomplete student participation. Only 73 of the 110 students enrolled in the course gave consent, possibly reflecting self-selection of students with more interest and experience with ultrasound. This is an inherent limitation of voluntary recruitment, making it difficult to eliminate. It is unclear how inclusion of these 37 students would have affected the study. It could be that inclusion of these students would have resulted in a greater difference between pre- and post-test scores in the near-peer group given near-peer instruction is different than what they had previously been exposed to. Alternatively, if they were less experienced with ultrasound and presumably randomized to both groups, their pre-test results may have lowered both groups’ scores slightly, but these students would have more potential to increase knowledge from the session resulting in higher differences between pre- and post-test results, and perhaps adding significance to these results. However, data collection was focused on comparing the effectiveness of instruction between the two groups as opposed to individual student ability. Additionally, prior ultrasound experience and pre-test scores were used to support randomization of students between the two groups. 

Prior to their third year at this institution, students had ultrasound sessions that covered some of the applications taught during Homeweek, and thus the assessments may partially reflect students’ ability to recall prior knowledge as opposed to instructor effectiveness during the sessions. However, ultrasound sessions during the first two years are dedicated to basic anatomy while the Homeweek sessions have a focus on clinical context, pathology, and evaluation through administration of an OSCE. While it would have been interesting to have a pre-session OSCE assessment to further support post-session improvement in image acquisition skills, it was not feasible due to student scheduling conflicts and resources required.

Results could have also been affected by potential teaching inconsistencies within the heterogeneous group of NP and faculty instructors. The basic teaching skills of the NP instructors likely sat on a spectrum of experience and ability. While they were recruited amongst a pool of experienced students and residents and were given an instructor guide to minimize potential variation, there were no in-person training sessions allowing them to practice teaching the content. While it may be of interest to evaluate the sub-groups of instructors, the sample size of this study limits the ability for any meaningful statistical analysis.

Our results reiterate conclusions of prior studies regarding NP instruction for skill-based education^4^, ^9^, ^10^, ^11^, ^12^ and support affirming attitudes towards NP instructor competency and ability to create a comfortable learning environment. However, our results fail to demonstrate the superiority of NP instructor student perception seen in some studies 14, 18, 19. One potential explanation is that senior medical students and residents recruited to be NP instructors may have lacked the confidence that faculty instructors have likely gained through years and repetition of teaching. Adding supplemental training alongside the incorporation of UME POCUS NP instruction may have a more positive effect on this 13.

Finally, our study focused on third-year medical student performance at a single institution with a robust longitudinal ultrasound curriculum and pool of NP instructors, and for this reason may not be generalizable to other institutions. Future studies should involve the collaboration of multiple institutions.

As development of UME POCUS curricula continues to expand, the incorporation of NP instructors should be integral to creating small group hands-on sessions with small student-to-instructor ratios for feasible effective POCUS teaching. 

## Conclusion

NP instructors are as effective as faculty instructors at teaching POCUS to third-year medical students at our institution. 

## Disclosures

### Funding: 

None

### Ethical approval: 

Study was approved by the University of Connecticut Health Center Institutional Review Board. Institutional Review Board study (number 20X-209-1) approved on 8/28/20. All participants gave consent for their evaluation data to be used for the purpose of publication.

### Disclaimers: 

None

## Other disclosures/Conflicts of interest:

Meghan Herbst is a question writer for the ABEM ultrasound focused practice designation examination, ACEP chair-elect, and has received travel support for ABEM ultrasound FPD question writing meetings.

## Supplementary Material

Supplementary Material File S1
