# Supplementary material for: Effectiveness of Near-Peer Versus Faculty Point-of-Care Ultrasound Instruction to Third-Year Medical Students
Source: POCUS J. 2022 Nov 21;7(2):239–44. doi: 10.24908/pocus.v7i2.15746 (PMC9983727; doi:10.24908/pocus.v7i2.15746)
Supplement: Supplementary Material File S1 [file pocusj-07-15746-s001.pdf]

POCUS Journal 2022; https://doi.org/10.24908/pocus.v7i2.15746

Appendix A

Ultrasound Session Instructor Guide

Goal: To teach MS3 basic bedside Point of care ultrasound (POCUS)

Learning Objectives:

1. Anatomy
    a. Cardiac
    b. Shoulder
    c. Ocular
    d. Thoracic
    e. Biliary
    f. Renal
2. Probe selection
    a. Linear
    b. Curvilinear
    c. Phase Array
3. Obtaining images
    a. Probe orientation and imaging planes
    b. Landmarks
    c. Machine knobology
4. Interpreting images
    a. Synthesis of all the above
    b. Prepared images from Dr. Herbst

5. Identifying pathology: Prepared images and clips from Dr. Herbst

Before you arrive

1. Preparation:
    - Read up on the cases you will be leading (in an attachment provided below)
    - Familiarize yourself with the objectives above, and adapt to your cases
2. Location: UConn Health Center, classroom B9 or Virtual Anatomy Lab, on the Main Level. Please contact Dr. Herbst if you need additional directions.

Day of

1. Bring:
    - Laptop (pull up the images and clips pertaining to your case)
2. Go to your room (VAL or B9)
    - Prep your station (gel, towels, gloves, Models)
3. Pre-scan your Model

During Sessions

1. Introduce yourself with your first name. **DO NOT PROVIDE YOUR CURRENT LEVEL OF TRAINING/POSITION AT WORK**
2. You have 30 minutes per group of students to finish a case
    - Make sure you meet the above learning objectives in that time frame
    - Every student in your group should have an opportunity to perform the point of care ultrasound examination.

After Sessions

1. We appreciate your help and time!
2. Leave everything as is. It will be taken care of.

Ultrasound Hands- On Cases

All **bolded** pathology will be sent to you in the form of anonymized clips via Dropbox. You are welcome to put them in a .ppt or .key file and show them on your phone or ipad/tablet (make sure they are set to auto-cycle).

Scenarios

1. OCULAR/THORACIC CASE

Read to students:

21yo male sustained physical assault to face and chest at a bar, including being punched to his left eye and having his head thrown face first into the ground. His chest hit the top of a chair on the way down. He is complaining of left eye pain and can't open his eye. He also has shortness of breath and right pleuritic chest pain. How would you approach his assessment with ultrasound?

Goals for students will be to perform an ultrasound of the eyeball, to indicate which frequency (ie, high), to identify the sonoanatomy visualized in a transverse plane (anterior chamber, posterior chamber, optic nerve sheath, lens), and to ask questions

about sonopathology (eg, is there a **vitreous hemorrhage**, **globe rupture**, **retrobulbar hematoma**, or **lens subluxation**). The students should also assess afferent pupillary function by shining a light in the contralateral eye and looking at the affected eye in a coronal plane. Students should also perform an ultrasound of the pleural line, looking for sliding, b-lines, z-lines, and assessing for a lung pulse. This patient has no ocular pathology but has a **lung point**.

2. BILIARY/RENAL CASE

Read to students:

42yo female develops right sided abd pain and vomiting. She does not exhibit tenderness when you press on her right abdomen, but states that (RUQ) is where her pain is. How would you approach her assessment with ultrasound?

Goal for students will be to perform an ultrasound of the gallbladder and kidney. They will likely start in the RUQ and identify the gallbladder with landmarks (they may not know landmarks, but be sure to point out the portal vein in sagittal with the neck of the gallbladder at approximately 1 o'clock from the PV). After scanning through in long axis, they can either look in short axis, or if short on time, let them know there are no stones, sonographic murphy's, wall thickening or PCF, and push them to look at another organ that can present with ruq pain and vomiting. Hopefully they will think to look at the kidney in coronal. Let them know there is right **moderate hydronephrosis** and if they look at the bladder in transverse, they can play with ureteral jets and you can show them a **right-sided uvj stone**.

3. LEFT SHOULDER PAIN CASE

Read to students:

50yo male with history of tobacco use, hypertension, and hypercholesterolemia developed left shoulder pain while shoveling snow in his driveway. How would you approach his assessment with ultrasound?

Goal for students will be to perform an ultrasound of the heart and left shoulder. They should be able to recognize the chambers in the parasternal long and short axis views (okay if they perform it with cardiology convention), and let them know everything looks okay. Might have to prompt with “negative troponin, negative EKG…look at the left shoulder”. With patient sitting and left arm supinated and adducted (forearm volar side up and resting on thigh) and the linear probe oriented transversely over the bicipital groove, the distal head of the biceps tendon can be visualized showing **fluid around the tendon** consistent with biceps tendonitis. You can also show the student how to view the shoulder joint posteriorly (ideally convex probe placed inferior to the scapular spine), if time.

Appendix B

Pre-Session Evaluation	Post-Session Evaluation	Categories
1. Where should the transducer indicator point on the patient when assessing the pleural line for lung sliding?<br>a. Anterior<br>b. Patient's left<br>c. Patient's right<br>d. Superior	1. Where should the transducer point on the patient when assessing for B-lines?<br>a. Anterior<br>b. Patient's left<br>c. Patient's right<br>d. Superior	physics/<br>**technique**/<br>sonoanatomy
2. Which of the following cardiac views at the level of the papillary muscles has the smallest number of chambers?<br>a. Apical<br>b. Parasternal long<br>c. Parasternal short<br>d. Subxiphoid	3. Which cardiac view demonstrates the mercedes benz sign?<br>a. Apical<br>b. Parasternal long<br>c. Parasternal short<br>d. Subxiphoid	physics/<br>technique/<br>**sonoanatomy**
3. Which biliary finding is the most sensitive for cholecystitis?<br>a. Biliary distension	4. Which cardiac finding is the most specific for acute pulmonary embolism?	clinical

b. Gallbladder wall thickening<br>c. Gallstones<br>d. Pericholecystic fluid	a. McConnell Sign<br>b. RV:LV ratio greater than 1<br>c. RV hypokinesis<br>d. Tricuspid regurgitation	
4. Which of the following cardiac views is best for evaluating a lateral wall motion abnormality?<br>a. Apical<br>b. Parasternal short<br>c. Subxiphoid<br>d. Suprasternal	2. Which of the following indicates depressed LV function?<br>a. 3mm E-point septal separation<br>b. 10mm E-point septal separation<br>c. 40% fractional shortening<br>d. 60% fractional shortening	clinical
5. Which of the following findings can be present in severe hydronephrosis but not in moderate hydronephrosis?<br>a. Cortical thinning<br>b. Increased number of dilated calyces<br>c. Larger kidney dimension<br>d. Pelvis dilatation	9. In the setting of an obstructing ureteral stone, dilation of which of the following structures is most likely to be visualized on ultrasound?<br>a. Calyces<br>b. Pelvis<br>c. Pyramids<br>d. Ureter	clinical
6. When performing point-of-care ultrasound, which structure can help	5. What ultrasound finding indicates the presence of a pneumothorax?	clinical

differentiate a pericardial effusion from a pleural effusion?<br>a. Ascending aorta<br>b. Descending aorta<br>c. Diaphragm<br>d. Liver	a. B-lines<br>b. Lung pulse<br>c. Lung point<br>d. Seashore sign	
7. Which is the best transducer to evaluate for a possible pneumothorax?<br>a. Curvilinear<br>b. Linear<br>c. Microconvex<br>d. Phased array	7. Which is the best transducer to evaluate a pericardial effusion?<br>a. Curvilinear<br>b. Linear<br>c. Microconvex<br>d. Phased array	physics/<br>**technique**/<br>sonoanatomy
8. Which mode best detects the directionality of blood flow?<br>a. A-mode<br>b. B-mode<br>c. Color doppler<br>d. Power doppler	8. Which mode best detects velocity of blood flow?<br>a. A-mode<br>b. B-mode<br>c. Color doppler<br>d. M-mode	**physics**/<br>technique/<br>sonoanatomy
9. In the subxiphoid view of the heart, the structure seen at the top of the screen is the:	6. When performing point-of-care biliary ultrasound, which structure is the best landmark for locating the	physics/<br>technique/<br>**sonoanatomy**

a. Left lung<br>b. Liver<br>c. Right lung<br>d. Spleen	gallbladder?<br>a. Hepatic vein<br>b. IVC<br>c. Pancreas<br>d. Portal vein	
10. Using a high frequency probe enables<br>a. Higher echogenicity of structures<br>b. Higher resolution of structures<br>c. Increased depth of penetration<br>d. Increased sensitivity of directionality of flow	10. Using a low frequency probe enables<br>a. Higher echogenicity of structures<br>b. Higher image resolution<br>c. Increased depth of penetration<br>d. Increased sensitivity of directionality of flow	**physics**/ technique/ sonoanatomy

Appendix C. Pre-Session Evaluation

Ultrasound Experience

- Do you have any ultrasound experience outside of the medical school curriculum? This may include shadowing clinical shifts with ultrasound faculty, participating in an ultrasound elective/ ILO, or volunteering to help with ultrasound courses.
    - Yes
    - No

Knowledge Questions

- Where should the transducer indicator point on the patient when assessing the pleural line for lung sliding?
    - Anterior
    - Patient’s left
    - Patient’s right
    - Superior
- Which of the following cardiac views at the level of the papillary muscles has the smallest number of chambers?
    - Apical
    - Parasternal long
    - Parasternal short
    - Subxiphoid
- Which biliary finding is the most sensitive for cholecystitis?
    - Biliary distension

  - Gallbladder wall thickening
  - Gallstones
  - Pericholecystic fluid
- Which of the following cardiac views is best for evaluating a lateral wall motion abnormality?
  - Apical
  - Parasternal short
  - Subxiphoid
  - Suprasternal
- Which of the following findings can be present in severe hydronephrosis but not in moderate hydronephrosis?
  - Cortical thinning
  - Increased number of dilated calyces
  - Larger kidney dimension
  - Pelvis dilatation
- When performing point-of-care ultrasound, which structure can help differentiate a pericardial effusion from a pleural effusion?
  - Ascending aorta
  - Descending aorta
  - Diaphragm
  - Liver
- Which is the best transducer to evaluate for a possible pneumothorax?
  - Curvilinear

  - Linear
  - Microconvex
  - Phased array
- Which mode best detects the directionality of blood flow?
  - A-mode
  - B-mode
  - Color doppler
  - Power doppler
- In the subxiphoid view of the heart, the structure seen at the top of the screen is the:
  - Left lung
  - Liver
  - Right lung
  - Spleen
- Using a high frequency probe enables
  - Higher echogenicity of structures
  - Higher resolution of structures
  - Increased depth of penetration
  - Increased sensitivity of directionality of flow

Appendix D. Post-Session Evaluation

Knowledge Questions

- Where should the transducer point on the patient when assessing for B-lines?
    - Anterior
    - Patient's left
    - Patient's right
    - Superior
- Which of the following indicates depressed LV function?
    - 3mm E-point septal separation
    - 10mm E-point septal separation
    - 40% fractional shortening
    - 60% fractional shortening
- Which cardiac view demonstrates the mercedes benz sign?
    - Apical
    - Parasternal long
    - Parasternal short
    - Subxiphoid
- Which cardiac finding is the most specific for acute pulmonary embolism?
    - McConnell Sign
    - RV:LV ratio greater than 1
    - RB hypokinesis
    - Tricuspid regurgitation
- What ultrasound finding indicates the presence of a pneumothorax?

  - B-lines
  - Lung pulse
  - Lung point
  - Seashore sign
- When performing point-of-care biliary ultrasound, which structure is the best landmark for locating the gallbladder?
  - Hepatic vein
  - IVC
  - Pancreas
  - Portal vein
- Which is the best transducer to evaluate a pericardial effusion?
  - Curvilinear
  - Linear
  - Microconvex
  - Phased array
- Which mode best detects velocity of blood flow?
  - A-mode
  - B-mode
  - Color doppler
  - M-mode
- In the setting of an obstructing ureteral stone, dilation of which of the following structures is most likely to be visualized on ultrasound?
  - Calyces

    - Pelvis
    - Pyramids
    - Ureter
- Using a low frequency probe enables
    - Higher echogenicity of structures
    - Higher image resolution
    - Increased depth of penetration
    - Increased sensitivity of directionality of flow

Likert Scale Questions

- Please indicate whether you agree or disagree with the following statement about your instructor for the following station. Assault vignette: evaluating for signs of ocular and thoracic trauma
    - Instructor was competent.
        - Strongly agree
        - Agree
        - Neutral
        - Disagree
        - Strongly disagree
    - The instructor created a comfortable environment to learn.
        - Strongly agree
        - Agree
        - Neutral
        - Disagree

        - Strongly disagree

- Please indicate whether you agree or disagree with the following statement about your instructor for the following station. Exertional left shoulder pain vignette: evaluating for signs of myocardial infarction and shoulder injury
    - Instructor was competent.
        - Strongly agree
        - Agree
        - Neutral
        - Disagree
        - Strongly disagree
    - The instructor created a comfortable environment to learn.
        - Strongly agree
        - Agree
        - Neutral
        - Disagree
        - Strongly disagree
- Please indicate whether you agree or disagree with the following statement about your instructor for the following station. Right sided abdominal pain vignette: evaluating for signs of cholecystitis and ureterolithiasis
    - Instructor was competent.
        - Strongly agree
        - Agree
        - Neutral

        - Disagree
        - Strongly disagree
    - The instructor created a comfortable environment to learn.
        - Strongly agree
        - Agree
        - Neutral
        - Disagree
        - Strongly disagree
- The ultrasound session was well organized.
    - Strongly agree
    - Agree
    - Neutral
    - Disagree
    - Strongly disagree
- My confidence in performing point of care ultrasound has improved through participation in the ultrasound session
    - Strongly agree
    - Agree
    - Neutral
    - Disagree
    - Strongly disagree

Appendix E. OSCE Instructor Guide

Goal: To examine MS3 POCUS knowledge after Homeweek POCUS sessions

Overview of Examination Objectives:

1. MCQ section
    a. To test for fundamental knowledge about POCUS and its applications
2. OSCE section (where you will be helping)
    a. To test for ability to obtain POCUS imaging
    b. To test for ability to interpret POCUS imaging

Before you arrive

1. Preparation: Familiarize yourself with the list of anatomy tested
2. Location: John Dempsey

Day of

1. Bring: Phone, phone charger
2. Prep your station: Gel, towels, gloves, Models w/ tegaderm on eye
3. Pre-scan your Model: You don't want to be surprised by anomalous anatomy

During Sessions

1. Introduce yourself and the Model
2. Introduce the testing parameters to the students:

a. Each student has 10 minutes to identify all 15 items on the list of anatomy
b. **DO NOT** show the list to the students. You can tell them there are 15 items in total, and that they are tested in their ability to find the anatomy
c. They either identify the anatomy correctly or they didn't. Binary.
d. Some items on the list will have a required imaging plane, some won't, let them know if there is a required view.
e. You will tell them "Find [anatomy]".
f. Inform them that when they have found the item in question, they must 'Freeze image' and point to and say "This is [anatomy]".
g. You will say "Okay. Find [next anatomy on the list]" whether or not they were correct, and move on. **DO NOT** let them know if they were correct or not.
h. Inform them that if they want to skip an item and come back to it later if they have time, allow them to return to it in the end, and continue moving along on the list.
i. Remember each student only has 10 minutes. There will be a reminder at the 2 minute mark.

3. Start OSCE Session (10 minutes each student)

OSCE 3rd Year Medical Students

NAME ___________________

Date and Time: _________________

**Please Identify**	Structure Identified Correctly	Structure NOT Identified Correctly
Vitreous chamber		
Lens		
Optic nerve		
Pleural line		
Rib shadows		
Parasternal long axis view, aortic outflow tract		
Apical 4-chamber view, left ventricle		
Subxiphoid view, right ventricle		

Interventricular septum		
Mitral valve		
Aortic valve		
Pelvis of left kidney in long axis		
Bladder in Sagittal		
Gallbladder neck in long axis		
Long head biceps tendon		
